# Trends in respiratory failure mortality in the United States from 1999 to 2023

**DOI:** 10.3389/fmed.2026.1718556

**Published:** 2026-02-23

**Authors:** Yunfei Shu, Suihan Xu, Biao Zeng, Zhenyu Yang, Jun Li, Wei Gao

**Affiliations:** 1 Department of Anesthesiology, The First Affiliated Hospital of Xi’an Jiaotong University, Xi’an, China; 2 Department of Anesthesiology, Mianyang Central Hospital, Mianyang, China

**Keywords:** CDC WONDER, disease burden, lung cancer, mortality, respiratory failure

## Abstract

**Background:**

The U.S. population is aging, accompanied by concurrent increases in the burden of respiratory failure. However, respiratory failure-related mortality trends among 45 years old adults have not been investigated. The purpose of this study was to assess the trends and regional differences in respiratory failure-related mortality among older adults in the United States.

**Methods:**

Data were obtained from the CDC WONDER database, encompassing death records of U.S. residents aged 45 years and older between 1999 and 2023. Cases were identified in which respiratory failure (ICD-10 codes J96.0, J96.1, and J96.9) was documented as the cause of death. We computed both crude and age-adjusted mortality rates (AAMR) per 100,000 population and employed Joinpoint regression to estimate annual percentage changes (APC) and average annual percentage change (AAPC). Additionally, a sensitivity analysis was conducted in which lung cancer was identified as the underlying cause of death and respiratory failure as a contributing cause, to assess the robustness of the conclusions.

**Results:**

A total of 186,075 respiratory failure-related deaths were recorded within the study period. The AAMR increased markedly from 3.71 in 1999 to 10.50 in 2023. The most pronounced upward trend occurred between 2005 and 2018 (APC: +7.96%; 95% CI: 7.44 to 8.93; *p* < 0.001). Specifically, mortality was higher among males than females (AAMR: 11.14 vs. 9.94) in 2023. Racial and ethnic disparities were evident, with non-Hispanic Black or African American individuals exhibiting the highest AAMR (14.07), compared to Hispanic individuals, who showed the lowest (5.96) in 2023. Geographically, rural/county areas experienced a significantly greater AAMR than large and medium/small metropolitan areas (12.27, 8.86, and 10.08, respectively) in 2020. Furthermore, distinct mortality trends were observed across various census regions. A sensitivity analysis-where lung cancer was identified as the underlying cause of death and respiratory failure as a contributing cause of death-confirmed this conclusion.

**Conclusion:**

Respiratory failure-related mortality has risen substantially over the study period, particularly after 2005, with significant inequalities observed across racial, gender, and geographic subgroups. These findings highlight the urgent need for targeted public health interventions to mitigate the increasing burden of these conditions.

## 1 Introduction

In the United States, population aging and the cumulative impacts of smoking, air pollution, chronic lung diseases (such as chronic obstructive pulmonary disease and interstitial lung disease), as well as coexisting cardiovascular conditions, have progressively rendered respiratory failure a common “final pathway” and a major contributor to mortality among middle-aged and older adults (1). As age increases, the decline in respiratory reserve combined with the accumulation of comorbidities significantly elevates both the risk of developing respiratory failure and the risk of death from it in individuals aged 45 years and older (2).

As lung reserve declines and multimorbidity accumulates, older adults disproportionately experience acute decompensations on the background of chronic respiratory diseases [e.g., chronic obstructive pulmonary disease (COPD) and interstitial lung disease] (3, 4)and cancer-related complications (e.g., airway obstruction and malignant pleural disease) (5). Prior data also suggest marked demographic and regional variation in respiratory-failure mortality, reflecting heterogeneity in socioeconomic conditions (e.g., poverty and healthcare access), environmental exposures (e.g., tobacco use and air pollution), and comorbid risk profiles (e.g., diabetes and obesity) (6).

The CDC WONDER database provides a standardized data source for long-term, nationwide, and stratified trend assessments, which provides comprehensive, accessible U.S. mortality data with county-level granularity and multidimensional demographic variables (7). As one of the most lethal adult malignancies, lung cancer not only shares overlapping risk factors with respiratory failure-such as smoking and air pollution-but also frequently leads to respiratory failure through progressive ventilation/perfusion disorders, post-obstruction infections, and malignant pleural lesions. Therefore, while estimating overall trends in respiratory failure as the primary cause of death, we performed a sensitivity analysis in which lung cancer was treated as the underlying cause of death. This analysis was designed to assess whether our findings were robust to alternative cause-of-death definitions and to minimize potential bias arising from misclassification.

Based on this, the present study employs Joinpoint regression to quantify temporal trends in respiratory failure-related mortality, identify demographic disparities-including those related to sex, race, urbanization level, and geographic region-and provide an evidence-based foundation for developing targeted interventions and public health policies aimed at optimizing clinical management.

## 2 Methods

### 2.1 Study setting and population

This descriptive investigation used mortality data from death certificates accessed through the CDC WONDER platform^1^ to analyze temporal trends in respiratory failure-related mortality, consistent with methodologies employed in previous studies (8). The primary aim was to assess mortality rates associated with respiratory failure among individuals aged 45 years and older between 1999 and 2023 in the underlying cause of death (UCD). This data set includes cause of death from death certificates for the 50 states and the District of Columbia (9). A sensitivity analysis was also conducted using respiratory failure-related lung cancer deaths where lung cancer was listed as the underlying cause of death and respiratory failure was listed as the contributing cause of death from the Multiple Cause of Death (MCD) public-access dataset (10). Respiratory failure-associated deaths were identified using ICD-10 codes J96.0 (acute respiratory failure), J96.1 (chronic respiratory failure), and J96.9 (unspecified respiratory failure). Lung cancer-related mortality was defined according to ICD-10 codes C34.0 (malignant neoplasm of the main bronchus), C34.1 (upper lobe of the lung or bronchus), C34.2 (middle lobe of the lung or bronchus), C34.3 (lower lobe of the lung or bronchus), C34.8 (overlapping sites of the bronchus and lung), and C34.9 (unspecified sites of the bronchus or lung).

The study incorporated all cases in which both conditions were documented on the death certificate. By systematically screening death certificates from UCD and MCD public medical records, we focused on identifying cases in which respiratory failure was listed as the cause of death. Since the analysis relied exclusively on de-identified, publicly accessible federal data, it was exempt from institutional review board (IRB) evaluation. Methodological transparency and rigor were maintained by complying with the STROBE guidelines for observational research.

### 2.2 Data abstraction

Mortality rates were disaggregated by major sociodemographic and geographical factors, such as gender, racial/ethnic background, degree of urbanization, and U.S. Census-designated regions (11). Racial and ethnic groups included Hispanic or Latino, non-Hispanic(NH) White, and NH Black or African American, aligning with categorical conventions frequently applied in earlier studies using the CDC WONDER data system. The distinction between city and country followed the 2013 NCHS Urban–Rural Classification Framework (12). Under this system, urban areas comprise large metropolitan regions (population ≥1 million) as well as small and medium metropolitan areas (population between 50,000 and 999,999), whereas rural counties are those with fewer than 50,000 inhabitants. Data on urbanicity levels for the years 2018–2023 is not available for download from the CDC WONDER database, as the National Center for Health Statistics (NCHS) has not yet released updated classifications based on the 2020 census. Therefore, this study was limited to investigating urbanization trends for the period 1999–2020. The country was partitioned into four geographic regions-Northeast, Midwest, South, and West-in accordance with U.S. Census Bureau criteria. Additionally, gender was categorized into male and female.

### 2.3 Statistical analysis

To characterize national patterns in respiratory failure-related mortality from 1999 to 2023, we computed crude and age-adjusted mortality rates (AAMRs) per 100,000 population by year, sex, race/ethnicity, state, and urban–rural status, each with 95% confidence intervals (CIs). Crude rates equaled respiratory failure-related deaths divided by the corresponding U.S. population for that year; AAMRs were obtained by direct standardization to the 2000 U.S. standard population. Temporal trends were evaluated with the National Cancer Institute’s Joinpoint Regression Program (Version 5.1.0), estimating annual percent change (APC) in AAMR and 95% CIs via log-linear models that detect inflection points (13).^2^ Additionally, a parallelism test was performed to determine whether temporal trends varied significantly across demographic subpopulations. A statistically significant result in this test suggests divergent trends in the average annual percent change (AAPC) among subgroups. An APC was deemed increasing or decreasing when the slope differed significantly from zero on two-tailed *t*-tests; statistical significance was set at *p* < 0.05.

## 3 Results

### 3.1 Overall trends in respiratory failure-related mortality among adults aged ≥45 years

Between 1999 and 2023, a total of 186,075 deaths associated with respiratory failure were recorded among individuals aged 45 years or older (Table 1; Supplementary Table 1). Over this period, the AAMR for respiratory failure-related deaths increased markedly, rising from 3.71 per 100,000 population (95% CI: 3.59–3.84) in 1999 to 10.50 per 100,000 population (95% CI: 10.33–10.67) in 2023. This corresponded to an average annual percent change (AAPC) of 4.67% (95% CI: 4.34–5.08; *p* < 0.001), indicating a sustained and substantial increase in respiratory failure-related mortality among middle-aged and older adults.

**Table 1:** Frequency and age-adjusted mortality rates per 100,000 in older adults with respiratory failure concomitantly stratified in adults aged 45 years and older by gender, race, census region and urbanization.

Subgroup	Deaths	Population	AAMR 1999 (95 % CI)	AAMR 2023 (95 % CI)	AAPC (95 % CI)
Overall	186075	3043725672	3.71 (3.59-3.84)	10.50 (10.33-10.67)	4.67 (4.34-5.08)
Gender					
Man	83520	1427661266	4.06 (3.85-4.28)	11.14 (10.88-11.41)	4.64 (4.25-5.04)
Woman	102555	1616064406	3.41 (3.26-3.56)	9.94 (9.72-10.15)	4.77 (4.43-5.21)
Race					
Hispanic	7614	308938513	1.83 (1.43-2.31)	5.96 (5.54-6.37)	7.21 (6.46-8.56)
NH White	149473	2245334172	3.60 (3.47-3.73)	11.20 (11.00-11.40)	4.94 (4.63-5.36)
NH Black or African American	25182	322117448	5.57 (5.03-6.10)	14.07 (13.44-14.69)	4.74 (4.18-5.28)
Census region of United States					
Northeast	48198	574121780	5.33 (5.02-5.65)	14.50 (14.05-14.95)	4.23 (3.89-4.62)
Midwest	41865	668417547	2.88 (2.66-3.10)	11.12 (10.75-11.49)	6.09 (5.70-6.61)
South	83433	1127663361	4.63 (4.40-4.87)	11.73 (11.44-12.01)	4.16 (3.83-4.58)
West	12579	673522984	1.15 (1.00-1.31)	4.32 (4.09-4.54)	5.99 (5.33-7.55)
Urbanization			AAMR 1999 (95 % CI)	AAMR 2020 (95 % CI)	AAPC (95 % CI)
Large	66029	1393581246	3.32 (3.16-3.49)	8.86 (8.64-9.07)	5.38 (4.80-6.31)
Medium/small	46529	794661897	4.26 (4.02-4.50)	10.08 (9.79-10.37)	4.21 (3.90-4.61)
Rural/counties	29599	434230530	3.64 (3.36-3.91)	12.27 (11.82-12.73)	6.03 (5.73-6.41)

A sensitivity analysis restricting lung cancer as the underlying cause of death, while considering respiratory failure as a contributing cause, revealed heterogeneous temporal patterns. Specifically, the AAMR declined from 1999 to 2006 (APC: −1.58%; 95% CI: −2.42 to −1.20; *p* < 0.05), followed by a plateau from 2006 to 2014 (APC: +0.11%; 95% CI: −0.63 to 0.49; *p* = 0.70). A significant increase was observed between 2014 and 2017 (APC: +2.08%; 95% CI: 1.09–2.68; *p* < 0.05), after which rates declined from 2017 to 2020 (APC: −1.59%; 95% CI: −2.26 to −0.64; *p* < 0.05). Notably, a renewed and pronounced upward trend emerged from 2020 to 2023 (APC: +2.44%; 95% CI: 1.60–4.15; *p* < 0.05) (Supplementary Figure S1).

### 3.2 Geographic disparities in respiratory failure-related mortality among middle-aged and older adults

**Figure 1:**
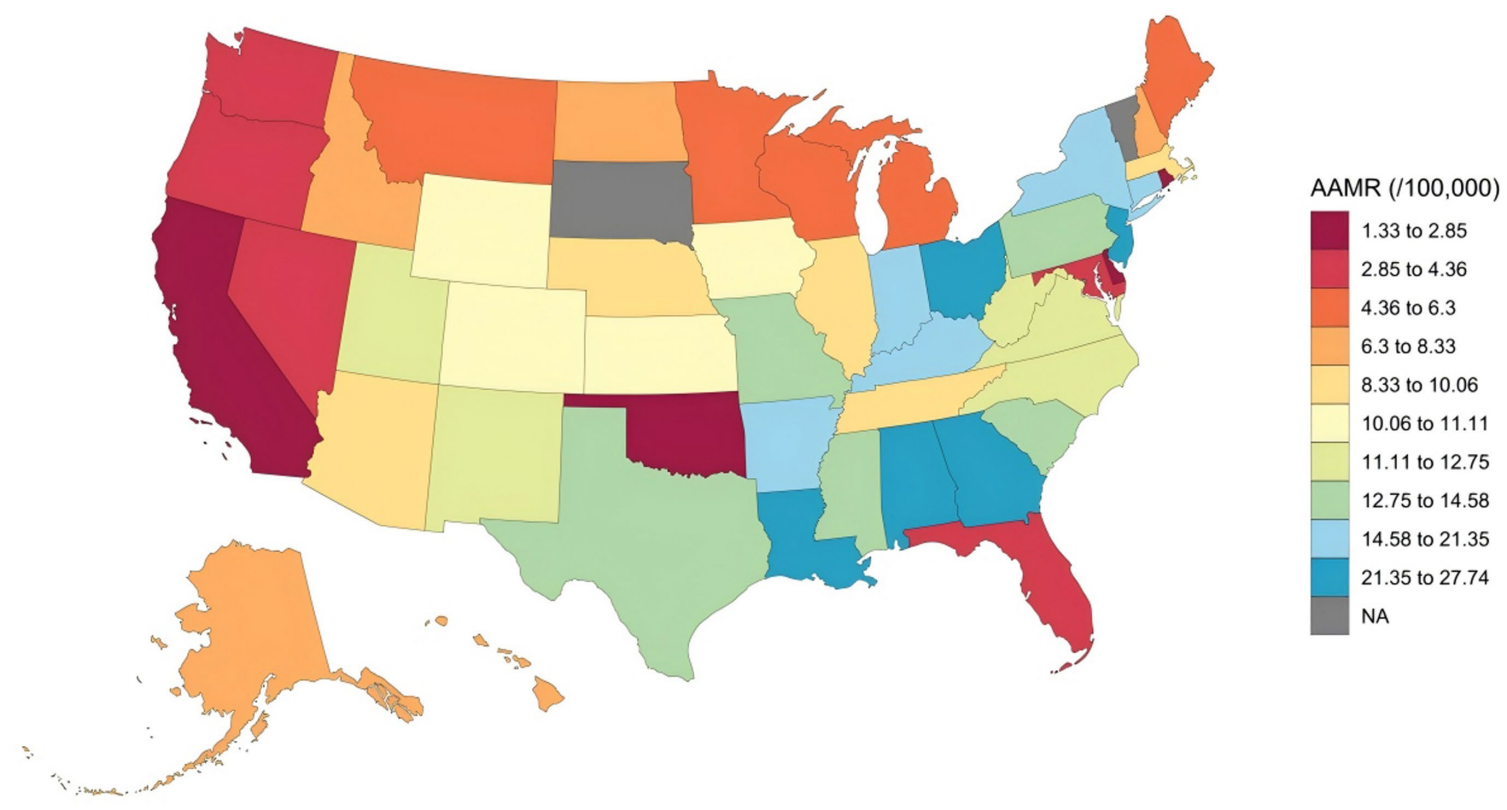
State-wise distribution of respiratory failure related age adjusted mortality rates in 2023. AAMR, age-adjusted mortality rates.

**Figure 2:**
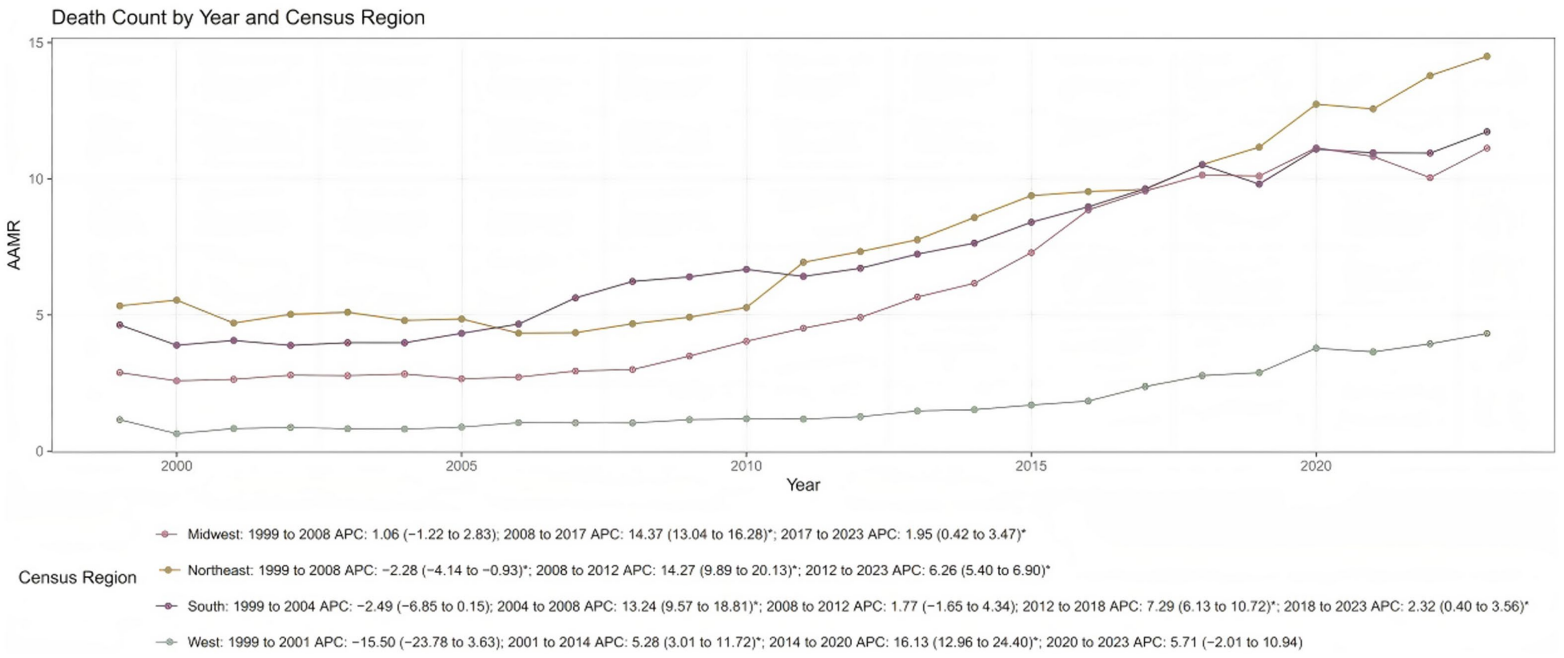
Trends in respiratory failure related age adjusted mortality rates by census region from 1999 to 2023. AAMR, age-adjusted mortality rates; APC, annual percent change.

Pronounced geographic disparities in respiratory failure-related mortality were observed across the United States. In 2023, state-level AAMRs varied widely, ranging from 1.33 per 100,000 population (95% CI: 1.15–1.50) in California to 27.74 per 100,000 population (95% CI: 26.12–29.35) in Georgia. States in the highest decile (e.g., Georgia, New Jersey, Louisiana, Alabama, and Ohio) had substantially higher AAMRs than those in the lowest decile (e.g., Nevada, Oregon, Oklahoma, and California) (Figure 1; Supplementary File). At the regional level, the Northeast experienced the highest respiratory failure-related mortality rate in 2023 (AAMR: 14.50; 95% CI: 14.05–14.95), whereas the West had the lowest rate (AAMR: 4.32; 95% CI: 4.09–4.54), followed by the South (AAMR: 11.73; 95% CI: 11.44–12.01) and Midwest (AAMR: 11.12; 95% CI: 10.75–11.49) (Table 1; Supplementary Table 3). In the Northeast, AAMRs declined modestly from 1999 to 2008 (APC: −2.28%; 95% CI: −4.14 to −0.93; *p* = 0.003), followed by a rapid increase from 2008 to 2012 (APC: +14.27%; 95% CI: 9.89–20.13; *p* = 0.001) and a continued rise from 2012 to 2023 (APC: +6.26%; 95% CI: 5.40–6.90; *p* = 0.002). In contrast, the West showed an initial sharp decline from 1999 to 2001, followed by successive periods of increase through 2023 (Figure 2).

**Figure 3:**
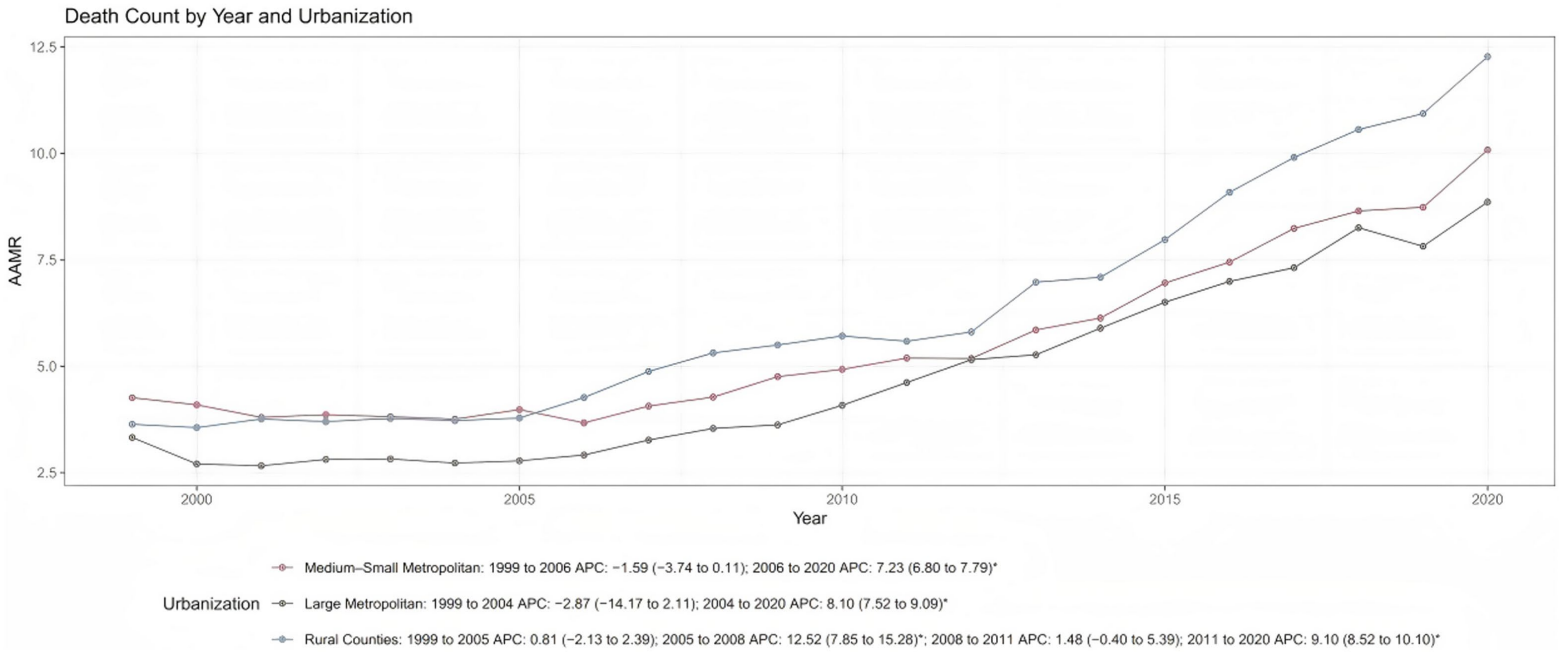
Trends in respiratory failure related age adjusted mortality rates by urbanization level from 1999 to 2020. AAMR: Age-adjusted mortality rates; APC: annual percent change.

Urban–rural disparities were also evident. In 2020, the highest AAMR was observed in rural counties (12.27; 95% CI: 11.82–12.73), while the lowest occurred in large metropolitan areas (8.86; 95% CI: 8.64–9.07), with intermediate rates in medium and small metropolitan areas (10.08; 95% CI: 9.79–10.37). Rural counties experienced a sustained increase in mortality beginning in the mid-2000s, with particularly rapid growth from 2011 to 2020 (APC: +9.10%; 95% CI: 8.52–10.10; *p* < 0.001). Similar but slightly attenuated upward trends were observed in metropolitan areas (Table 1; Figure 3; Supplementary Table 4).

### 3.3 Sex-specific trends in respiratory failure-related mortality among older adults

**Figure 4:**
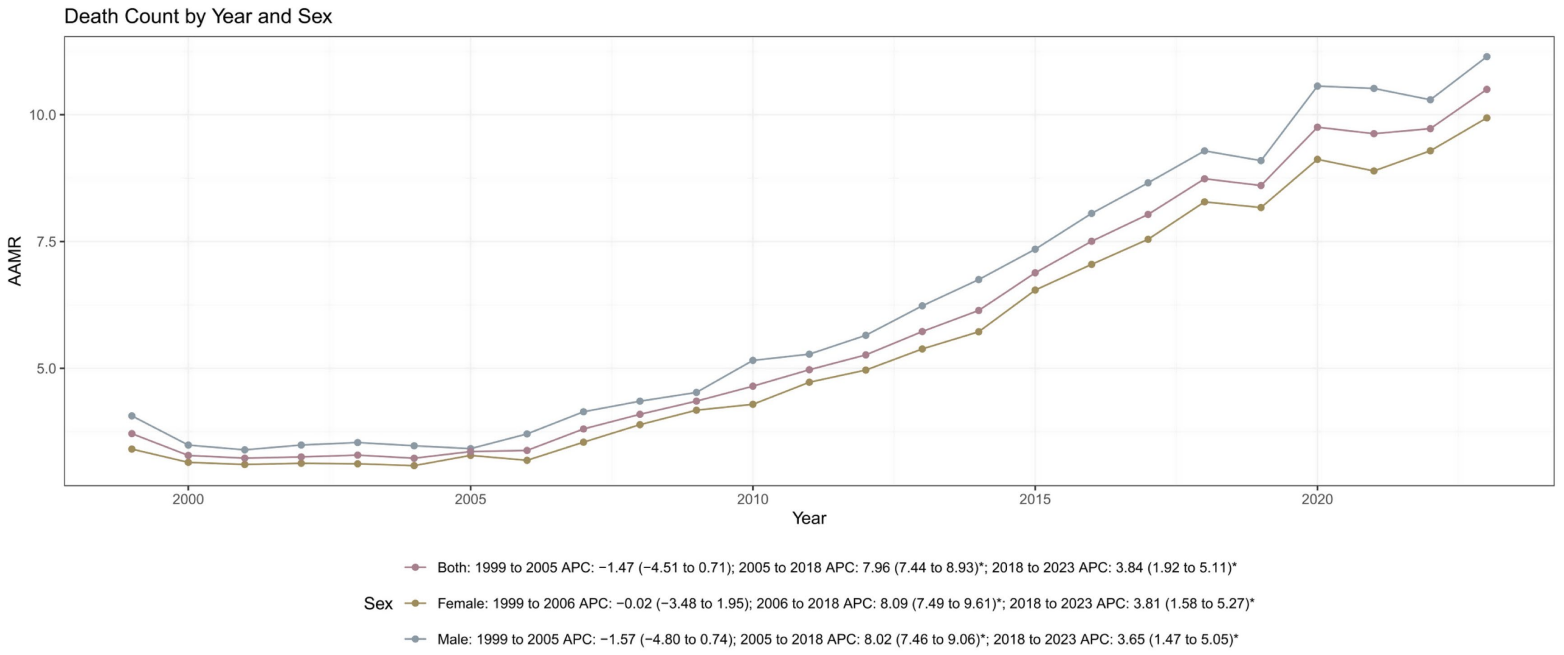
Trends in respiratory failure related age adjusted mortality rates by gender from 1999 to 2023. AAMR, age-adjusted mortality rates; APC, annual percent change.

Overall respiratory failure-related mortality increased in both sexes over the study period, although temporal patterns differed by gender. Among males, the AAMR increased from 4.06 (95% CI: 3.85–4.28) in 1999 to 11.14 (95% CI: 10.88–11.41) in 2023. Mortality rates declined slightly between 1999 and 2005, followed by a pronounced increase from 2005 to 2018 (APC: +8.02%; 95% CI: 7.46–9.06; *p* < 0.001), and a continued rise from 2018 to 2023 (Table 1; Figure 4; Supplementary Table 1).

Among females, the AAMR rose from 3.41 (95% CI: 3.26–3.56) in 1999 to 9.94 (95% CI: 9.72–10.15) in 2023. Joinpoint analysis revealed a stable period from 1999 to 2006, followed by a sharp increase from 2006 to 2018 (APC: +8.09%; 95% CI: 7.49–9.61; *p* < 0.001), and a slower but sustained increase thereafter. Although mortality rates remained higher among males throughout the study period, the rate of increase was slightly greater among females (AAPC: +4.77%; 95% CI: 4.43–5.21) than among males (AAPC: +4.64%; 95% CI: 4.25–5.04), indicating a narrowing sex gap in respiratory failure-related mortality (Table 1).

### 3.4 Racial and ethnic disparities in respiratory failure-related mortality

Substantial racial and ethnic disparities in respiratory failure-related mortality were observed among middle-aged and older adults. Among Hispanic or Latino individuals, the AAMR increased steadily from 1.83 (95% CI: 1.43–2.31) in 1999 to 5.96 (95% CI: 5.54–6.37) in 2023, with a consistent upward trend throughout the study period (APC: +7.21%; 95% CI: 6.46–8.56; *p* < 0.001). Among non-Hispanic White individuals, the AAMR rose from 3.60 (95% CI: 3.47–3.73) in 1999 to 11.20 (95% CI: 11.00–11.40) in 2023. Mortality rates remained relatively stable until 2005, increased sharply from 2005 to 2018, and then entered a phase of slower growth thereafter.

**Figure 5:**
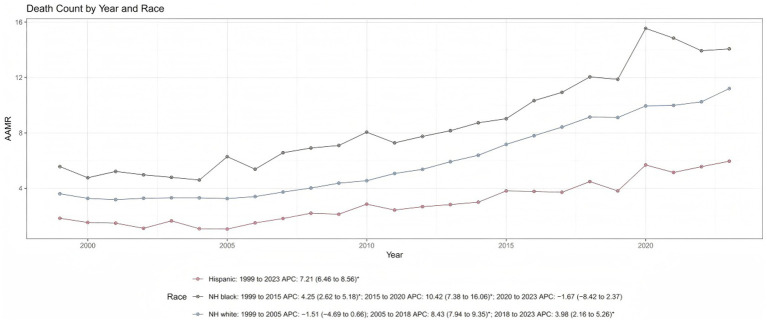
Trends in respiratory failure related age adjusted mortality rates by race from 1999 to 2023. AAMR, age-adjusted mortality rates; APC, annual percent change.

Non-Hispanic Black or African American individuals consistently exhibited the highest respiratory failure-related mortality rates across all years, with the AAMR increasing from 5.57 (95% CI: 5.03–6.10) in 1999 to 14.07 (95% CI: 13.44–14.69) in 2023. Following a moderate increase from 1999 to 2015, mortality rose sharply between 2015 and 2020 but showed a nonsignificant decline from 2020 to 2023 (Table 1; Figure 5; Supplementary Table 2).

## 4 Discussion

This study analyzed mortality trends related to respiratory failure among adults aged 45 years and older in the U.S. from 1999 to 2023 using data from the CDC WONDER database. The findings indicated that age-adjusted mortality rates experienced a pronounced increase between 2005 and 2018, followed by a steady rise from 2018 to 2023. These trends were consistent across the sensitivity analysis and all demographic subgroups. Specifically, males consistently exhibited higher AAMRs than females. Among racial groups, NH Black individuals had the highest mortality rates, followed by NH White individuals and Hispanics. Geographically, rural and counties experienced higher mortality rates than metropolitan areas, with the Northeast region reporting the highest AAMRs. Notably, states in the 90th percentile of AAMRs had substantially higher mortality rates than those in the 10th percentile.

With the intensification of population aging, the proportion of individuals aged over 45 years has steadily increased. This population is more likely to suffer from chronic conditions, such as COPD and cardiovascular diseases, and often presents with multiple coexisting comorbidities. These interacting conditions place an increased burden on the respiratory system, thereby elevating the risk of respiratory failure (14). Furthermore, the management of chronic diseases, particularly COPD, is especially challenging in older adults. In addition, the uneven distribution of healthcare resources limits timely access to adequate medical care for some elderly individuals, which may lead to delayed diagnosis and treatment, further exacerbating disease progression and mortality risk (15). Additionally, although the overall air quality has improved, the elderly have a lower tolerance for air pollutants, and prolonged exposure still increases the risk of respiratory diseases (16). At the same time, the immune system of the elderly weakens, making them more susceptible to respiratory diseases such as influenza and pneumonia. These infections are common causes of respiratory failure (17). Some elderly individuals also cannot receive timely medical care due to economic reasons, and lack family and social support, making it difficult for them to effectively manage their health conditions (18).

The results further underscore significant disparities among racial and ethnic groups, with Hispanic or Latino adults demonstrating the lowest incidence of adverse events associated with respiratory failure, whereas non-Hispanic Black or African American adults exhibited the highest rates. A confluence of factors likely contributes to these observed differences. Primarily, variations in baseline health play a pivotal role. Non-Hispanic Black or African American individuals disproportionately bear a higher burden of chronic conditions that are established risk factors for respiratory failure, including hypertension, diabetes, and chronic kidney disease. National data indicate that non-Hispanic Black adults have a significantly higher prevalence of hypertension compared with the general adult population and are more frequently diagnosed with diabetes and diabetes-related end-stage renal disease than other racial/ethnic groups. Additionally, racial/ethnic disparities in multimorbidity-defined as the coexistence of multiple chronic conditions-have been documented in U.S. populations, with non-Hispanic Black individuals showing greater accumulation of chronic diseases over time compared with non-Hispanic White and Hispanic groups (19, 20). Socioeconomic determinants also represent a crucial dimension. Ethnic minority groups are more likely to experience elevated poverty levels, constrained access to healthcare services, and lower rates of health insurance coverage-all of which may lead to inadequate disease management and delays in receiving timely medical care (21, 22). Furthermore, inequities in healthcare quality represent another important contributor. Evidence suggests that minority patients may receive substandard care in critical areas, including intensive care and respiratory failure management, manifesting as delays in symptom recognition, diagnostic evaluation, and appropriate treatment intensity (23, 24).

In addition, we observed significant geographical disparities in respiratory failure-related mortality among middle-aged and older adults, with the Northeastern United States exhibiting higher rates compared to other regions in2023. This phenomenon is likely the result of interacting factors, including demographic structure, environmental conditions, socioeconomic status, and healthcare accessibility. As a traditionally industrial region with an older population, the Northeast bears a heavier burden of chronic underlying diseases and has a historical legacy of air pollution (25, 26). Previous studies have shown that colder climates are associated with an increased burden of respiratory diseases, which may exacerbate respiratory conditions and contribute to worse clinical outcomes (27). In addition, existing literature indicates that, even in regions with high-quality medical resources, pronounced intraregional socioeconomic disparities can limit timely and adequate access to healthcare for vulnerable populations. Such disparities may result in delays in treatment for respiratory failure or differences in end-of-life care strategies. These observations represent general patterns reported in prior research rather than findings specific to the present study. The convergence of these multifactorial influences collectively elevates the risk of respiratory failure-related mortality in this area. Our findings highlight the necessity of conducting large-scale population studies in these regions to identify the predominant drivers behind such disparities.

Nonmetropolitan areas were found to bear a substantially higher burden of respiratory failure-related mortality among middle-aged and older adults compared with metropolitan areas. Over the past decade, age-adjusted mortality rates associated with respiratory failure have increased steadily in both settings, with a notably more rapid rise observed in nonmetropolitan regions. This widening disparity likely reflects a complex interplay of structural, clinical, and population-level factors. A major contributor is the inequitable distribution of healthcare resources. Nonmetropolitan areas frequently face limited emergency medical service coverage, reduced availability of intensive care units, and shortages of pulmonary and critical care specialists. These constraints may lead to delays in diagnosis and initiation of appropriate treatment, which is particularly detrimental in time-sensitive conditions such as respiratory failure (28). In addition, differences in population structure and baseline health status may further exacerbate this disparity. Nonmetropolitan populations tend to be older and have a higher prevalence of chronic conditions-including COPD, obesity, diabetes, and cardiovascular disease-that are well-established risk factors for severe and fatal respiratory failure (29, 30). From a clinical and preventive perspective, behavioral and preventive factors, such as smoking exposure and vaccination status, may also play an important role in shaping these geographic differences. Smoking prevalence remains consistently higher in nonmetropolitan areas, contributing to chronic lung disease and reduced respiratory reserve, while lower uptake of influenza and pneumococcal vaccinations may increase susceptibility to severe respiratory infections that precipitate respiratory failure. However, individual-level data on smoking history and vaccination status were not available in the mortality database used for this study, precluding direct evaluation of their contributions. Future studies incorporating such clinical and behavioral data are warranted to better elucidate modifiable risk factors and to inform targeted prevention strategies in high-risk nonmetropolitan populations.

This study has several limitations that should be acknowledged (11, 31). First, reliance on ICD coding and death certificate data introduces the potential for misclassification or underreporting of respiratory failure as a cause of death. Second, the database lacks detailed clinical information necessary for more precise phenotyping, including vital signs, laboratory test results, arterial blood gas measurements, and pulmonary function parameters. Third, data on specific medical interventions-particularly treatment strategies and intensity of care for respiratory failure-were not available, limiting the ability to assess the impact of clinical management on mortality outcomes. In addition, although this study provides a comprehensive national-level analysis across U. S. states, it does not account for state-specific social, political, or health policy measures implemented over the long study period, such as differences in healthcare organization, insurance coverage, or public health initiatives. These contextual variations may have influenced respiratory failure-related mortality trends and could not be explicitly evaluated in the present analysis. Importantly, the study period encompasses the COVID-19 pandemic, which substantially altered patterns of respiratory disease, healthcare utilization, and mortality. Although our analyses capture overall temporal changes during this period, we did not perform a dedicated COVID-19-specific analysis stratified by mortality, sex, race/ethnicity, or public health interventions. The absence of detailed information on SARS-CoV-2 infection status, vaccination history, pandemic-related healthcare disruptions, and evolving treatment protocols limits our ability to disentangle the direct and indirect effects of COVID-19 on respiratory failure-related mortality trends. Finally, the lack of individual-level data on social determinants of health-such as income, educational attainment, and healthcare accessibility-may further influence the interpretation of observed disparities. These limitations should be considered when interpreting the findings.

## 5 Conclusion

The data indicate that the AAMR related to respiratory failure among middle-aged and older adults has shown a consistent upward trend. Notably, the highest AAMR values were observed among non-Hispanic Black or African American individuals, males, residents of the Northeastern United States, and those living in nonmetropolitan areas. To reduce respiratory failure-related mortality, it is imperative to enhance prevention and treatment efforts targeting respiratory failure in the middle-aged and elderly population.

## Data Availability

The original contributions presented in the study are included in the article/Supplementary material, further inquiries can be directed to the corresponding authors.

## Footnotes

1 https://wonder.cdc.gov/.

2 https://surveillance.cancer.gov/joinpoint/download.
