# Supplementary material for: Trends in respiratory failure mortality in the United States from 1999 to 2023
Source: Front Med (Lausanne). 2026 Feb 23;13:1718556. doi: 10.3389/fmed.2026.1718556 (PMC12969284; doi:10.3389/fmed.2026.1718556)

**Manuscript title:** “Trends in Respiratory Failure Mortality in the United States from 1999 to 2023”

**Table 1.** Respiratory failure–related age-adjusted mortality rates per 100,000, stratified by Sex in adults aged 45 years and older in the United States, 1999 to 2023.

| Year | **Age-Adjusted Mortality Rate (95% CI)** | | |
| --- | --- | --- | --- |
|  | **Overall** | **Male** | **Female** |
| 1999 | 3.71 (3.59-3.84) | 4.06 (3.85-4.28) | 3.41 (3.26-3.56) |
| 2000 | 3.28 (3.17-3.40) | 3.49 (3.29-3.68) | 3.15 (3.00-3.29) |
| 2001 | 3.23 (3.12-3.34) | 3.39 (3.20-3.58) | 3.10 (2.96-3.25) |
| 2002 | 3.25 (3.14-3.37) | 3.49 (3.30-3.68) | 3.13 (2.99-3.27) |
| 2003 | 3.29 (3.18-3.40) | 3.54 (3.35-3.73) | 3.12 (2.98-3.26) |
| 2004 | 3.23 (3.12-3.34) | 3.47 (3.29-3.66) | 3.08 (2.94-3.22) |
| 2005 | 3.36 (3.25-3.47) | 3.42 (3.23-3.60) | 3.28 (3.14-3.43) |
| 2006 | 3.38 (3.27-3.49) | 3.71 (3.52-3.89) | 3.19 (3.05-3.32) |
| 2007 | 3.80 (3.69-3.92) | 4.14 (3.95-4.34) | 3.54 (3.40-3.69) |
| 2008 | 4.09 (3.97-4.21) | 4.35 (4.16-4.55) | 3.89 (3.74-4.04) |
| 2009 | 4.35 (4.23-4.48) | 4.53 (4.33-4.72) | 4.17 (4.02-4.33) |
| 2010 | 4.65 (4.52-4.77) | 5.16 (4.95-5.36) | 4.29 (4.13-4.45) |
| 2011 | 4.97 (4.84-5.10) | 5.28 (5.07-5.49) | 4.73 (4.56-4.89) |
| 2012 | 5.26 (5.13-5.39) | 5.65 (5.44-5.86) | 4.97 (4.80-5.13) |
| 2013 | 5.73 (5.59-5.86) | 6.23 (6.01-6.45) | 5.38 (5.21-5.55) |
| 2014 | 6.14 (6.00-6.28) | 6.75 (6.53-6.97) | 5.72 (5.55-5.89) |
| 2015 | 6.88 (6.74-7.03) | 7.35 (7.12-7.58) | 6.54 (6.36-6.73) |
| 2016 | 7.51 (7.36-7.65) | 8.05 (7.82-8.29) | 7.05 (6.86-7.24) |
| 2017 | 8.03 (7.88-8.19) | 8.66 (8.41-8.90) | 7.54 (7.35-7.74) |
| 2018 | 8.74 (8.58-8.89) | 9.29 (9.04-9.54) | 8.28 (8.08-8.48) |
| 2019 | 8.60 (8.45-8.76) | 9.09 (8.85-9.34) | 8.17 (7.97-8.37) |
| 2020 | 9.75 (9.59-9.92) | 10.56 (10.31-10.82) | 9.12 (8.91-9.33) |
| 2021 | 9.63 (9.46-9.79) | 10.52 (10.26-10.78) | 8.89 (8.68-9.10) |
| 2022 | 9.73 (9.57-9.88) | 10.29 (10.04-10.55) | 9.29 (9.08-9.50) |
| 2023 | 10.50 (10.33-10.67) | 11.14 (10.88-11.41) | 9.94 (9.72-10.15) |

**Table 2.** Respiratory failure –related age-adjusted mortality rates per 100,000, stratified by Race in adults aged 45 years and older in the United States, 1999 to 2023.

| Year | **Age-Adjusted Mortality Rate (95% CI)** | | |
| --- | --- | --- | --- |
|  | **NH Black or African American** | **NH White** | **Hispanic or Latino** |
| 1999 | 5.57 (5.03-6.10) | 3.60 (3.47-3.73) | 1.83 (1.43-2.31) |
| 2000 | 4.76 (4.27-5.25) | 3.27 (3.15-3.39) | 1.53 (1.17-1.96) |
| 2001 | 5.22 (4.71-5.72) | 3.18 (3.06-3.30) | 1.48 (1.14-1.89) |
| 2002 | 4.97 (4.48-5.46) | 3.28 (3.16-3.40) | 1.11 (0.82-1.46) |
| 2003 | 4.79 (4.31-5.27) | 3.31 (3.19-3.43) | 1.65 (1.31-2.05) |
| 2004 | 4.60 (4.13-5.06) | 3.31 (3.19-3.43) | 1.08 (0.81-1.40) |
| 2005 | 6.28 (5.74-6.82) | 3.25 (3.13-3.37) | 1.05 (0.81-1.35) |
| 2006 | 5.38 (4.89-5.86) | 3.40 (3.28-3.52) | 1.50 (1.20-1.86) |
| 2007 | 6.57 (6.03-7.10) | 3.73 (3.60-3.86) | 1.81 (1.47-2.16) |
| 2008 | 6.91 (6.37-7.45) | 4.02 (3.88-4.15) | 2.20 (1.83-2.56) |
| 2009 | 7.09 (6.54-7.64) | 4.37 (4.23-4.50) | 2.13 (1.78-2.47) |
| 2010 | 8.05 (7.48-8.62) | 4.55 (4.41-4.68) | 2.85 (2.46-3.25) |
| 2011 | 7.28 (6.74-7.81) | 5.07 (4.92-5.21) | 2.43 (2.08-2.77) |
| 2012 | 7.75 (7.20-8.29) | 5.37 (5.22-5.51) | 2.67 (2.31-3.02) |
| 2013 | 8.16 (7.62-8.70) | 5.92 (5.77-6.07) | 2.82 (2.47-3.17) |
| 2014 | 8.73 (8.18-9.28) | 6.39 (6.23-6.54) | 3.00 (2.65-3.34) |
| 2015 | 9.02 (8.48-9.57) | 7.17 (7.00-7.34) | 3.81 (3.43-4.19) |
| 2016 | 10.33 (9.75-10.91) | 7.80 (7.62-7.97) | 3.77 (3.40-4.15) |
| 2017 | 10.93 (10.34-11.51) | 8.42 (8.24-8.60) | 3.71 (3.35-4.07) |
| 2018 | 12.04 (11.44-12.65) | 9.14 (8.96-9.33) | 4.48 (4.10-4.87) |
| 2019 | 11.87 (11.28-12.46) | 9.11 (8.93-9.29) | 3.81 (3.46-4.15) |
| 2020 | 15.55 (14.89-16.21) | 9.94 (9.76-10.13) | 5.68 (5.27-6.09) |
| 2021 | 14.84 (14.19-15.50) | 9.98 (9.79-10.18) | 5.14 (4.75-5.53) |
| 2022 | 13.93 (13.31-14.56) | 10.24 (10.05-10.43) | 5.56 (5.16-5.96) |
| 2023 | 14.07 (13.44-14.69) | 11.20 (11.00-11.40) | 5.96 (5.54-6.37) |

**Table 3.** Respiratory failure –related age-adjusted mortality rates per 100,000, stratified by Census region in adults aged 45 years and older in the United States, 1999 to 2023.

| Year | **Age-Adjusted Mortality Rate (95% CI)** | | | |
| --- | --- | --- | --- | --- |
|  | **Northeast** | **Midwest** | **South** | **West** |
| 1999 | 5.33 (5.02-5.65) | 2.88 (2.66-3.10) | 4.63 (4.40-4.87) | 1.15 (1.00-1.31) |
| 2000 | 5.54 (5.22-5.86) | 2.58 (2.37-2.79) | 3.88 (3.67-4.10) | 0.64 (0.52-0.75) |
| 2001 | 4.70 (4.41-4.99) | 2.63 (2.43-2.84) | 4.06 (3.85-4.28) | 0.83 (0.70-0.95) |
| 2002 | 5.02 (4.72-5.32) | 2.79 (2.58-3.00) | 3.88 (3.67-4.09) | 0.87 (0.74-1.00) |
| 2003 | 5.10 (4.80-5.40) | 2.77 (2.56-2.98) | 3.98 (3.77-4.19) | 0.82 (0.70-0.94) |
| 2004 | 4.79 (4.50-5.09) | 2.83 (2.62-3.04) | 3.98 (3.77-4.18) | 0.81 (0.69-0.93) |
| 2005 | 4.85 (4.56-5.14) | 2.65 (2.45-2.86) | 4.32 (4.11-4.54) | 0.88 (0.75-1.00) |
| 2006 | 4.33 (4.05-4.60) | 2.71 (2.51-2.92) | 4.66 (4.44-4.88) | 1.04 (0.91-1.18) |
| 2007 | 4.34 (4.07-4.62) | 2.94 (2.73-3.15) | 5.63 (5.39-5.86) | 1.03 (0.90-1.17) |
| 2008 | 4.67 (4.39-4.96) | 2.99 (2.78-3.21) | 6.23 (5.98-6.48) | 1.03 (0.90-1.16) |
| 2009 | 4.92 (4.63-5.20) | 3.49 (3.26-3.72) | 6.40 (6.15-6.64) | 1.15 (1.02-1.29) |
| 2010 | 5.27 (4.97-5.57) | 4.03 (3.79-4.28) | 6.67 (6.42-6.92) | 1.19 (1.05-1.32) |
| 2011 | 6.93 (6.59-7.27) | 4.51 (4.26-4.77) | 6.41 (6.17-6.65) | 1.18 (1.04-1.31) |
| 2012 | 7.33 (6.98-7.67) | 4.91 (4.64-5.17) | 6.71 (6.47-6.96) | 1.26 (1.12-1.40) |
| 2013 | 7.76 (7.41-8.11) | 5.66 (5.37-5.94) | 7.23 (6.98-7.48) | 1.47 (1.32-1.62) |
| 2014 | 8.57 (8.21-8.94) | 6.16 (5.87-6.45) | 7.64 (7.38-7.89) | 1.52 (1.37-1.67) |
| 2015 | 9.38 (9.00-9.76) | 7.29 (6.97-7.60) | 8.40 (8.14-8.66) | 1.69 (1.54-1.84) |
| 2016 | 9.53 (9.14-9.91) | 8.85 (8.51-9.20) | 8.97 (8.70-9.24) | 1.84 (1.68-1.99) |
| 2017 | 9.61 (9.23-9.99) | 9.55 (9.20-9.90) | 9.63 (9.36-9.90) | 2.37 (2.19-2.54) |
| 2018 | 10.52 (10.12-10.91) | 10.14 (9.77-10.50) | 10.51 (10.23-10.79) | 2.77 (2.58-2.96) |
| 2019 | 11.16 (10.76-11.56) | 10.10 (9.74-10.46) | 9.80 (9.54-10.07) | 2.88 (2.69-3.07) |
| 2020 | 12.74 (12.31-13.17) | 11.13 (10.76-11.51) | 11.09 (10.81-11.37) | 3.78 (3.57-3.99) |
| 2021 | 12.56 (12.13-13.00) | 10.82 (10.44-11.19) | 10.95 (10.67-11.24) | 3.64 (3.43-3.85) |
| 2022 | 13.79 (13.35-14.23) | 10.04 (9.69-10.39) | 10.94 (10.66-11.22) | 3.94 (3.72-4.15) |
| 2023 | 14.50 (14.05-14.95) | 11.12 (10.75-11.49) | 11.73 (11.44-12.01) | 4.32 (4.09-4.54) |

| Year | **Age-Adjusted Mortality Rate (95% CI)** | | |
| --- | --- | --- | --- |
|  | **Large Metropolitan** | **Medium/Small Metropolitan** | **Rural/Counties** |
| 1999 | 3.32 (3.16-3.49) | 4.26 (4.02-4.50) | 3.64 (3.36-3.91) |
| 2000 | 2.70 (2.56-2.85) | 4.09 (3.86-4.32) | 3.56 (3.28-3.83) |
| 2001 | 2.66 (2.52-2.81) | 3.80 (3.58-4.02) | 3.76 (3.48-4.04) |
| 2002 | 2.81 (2.66-2.96) | 3.86 (3.64-4.08) | 3.70 (3.42-3.97) |
| 2003 | 2.82 (2.68-2.97) | 3.81 (3.59-4.03) | 3.77 (3.50-4.05) |
| 2004 | 2.73 (2.58-2.87) | 3.76 (3.54-3.97) | 3.72 (3.45-3.99) |
| 2005 | 2.78 (2.64-2.92) | 3.98 (3.76-4.20) | 3.78 (3.51-4.06) |
| 2006 | 2.91 (2.77-3.06) | 3.67 (3.46-3.87) | 4.26 (3.97-4.55) |
| 2007 | 3.27 (3.11-3.42) | 4.06 (3.85-4.28) | 4.88 (4.57-5.19) |
| 2008 | 3.54 (3.38-3.69) | 4.27 (4.06-4.49) | 5.31 (4.99-5.63) |
| 2009 | 3.62 (3.47-3.78) | 4.75 (4.53-4.98) | 5.50 (5.17-5.82) |
| 2010 | 4.08 (3.92-4.25) | 4.92 (4.70-5.15) | 5.71 (5.38-6.04) |
| 2011 | 4.62 (4.44-4.79) | 5.19 (4.96-5.42) | 5.59 (5.26-5.91) |
| 2012 | 5.15 (4.97-5.33) | 5.18 (4.95-5.41) | 5.80 (5.48-6.13) |
| 2013 | 5.27 (5.09-5.45) | 5.85 (5.61-6.09) | 6.97 (6.62-7.33) |
| 2014 | 5.89 (5.70-6.08) | 6.13 (5.88-6.37) | 7.09 (6.73-7.44) |
| 2015 | 6.50 (6.31-6.70) | 6.95 (6.69-7.21) | 7.97 (7.60-8.35) |
| 2016 | 6.99 (6.79-7.19) | 7.44 (7.18-7.71) | 9.08 (8.69-9.48) |
| 2017 | 7.31 (7.11-7.51) | 8.24 (7.96-8.51) | 9.90 (9.49-10.31) |
| 2018 | 8.25 (8.04-8.46) | 8.65 (8.37-8.92) | 10.56 (10.14-10.98) |
| 2019 | 7.82 (7.61-8.02) | 8.73 (8.46-9.01) | 10.93 (10.50-11.36) |
| 2020 | 8.86 (8.64-9.07) | 10.08 (9.79-10.37) | 12.27 (11.82-12.73) |

**Table 4.** Respiratory failure–related age-adjusted mortality rates per 100,000, stratified by urbanization in adults aged 45 years and older in the United States, 1999 to 2020.

**Figure S1.** Age-adjusted mortality rates (AAMRs) per 100,000 in adults aged 45 years and older in the United States from 1999 to 2023, with lung cancer as a underlying cause of death and Respiratory Failure the contributing cause of death.


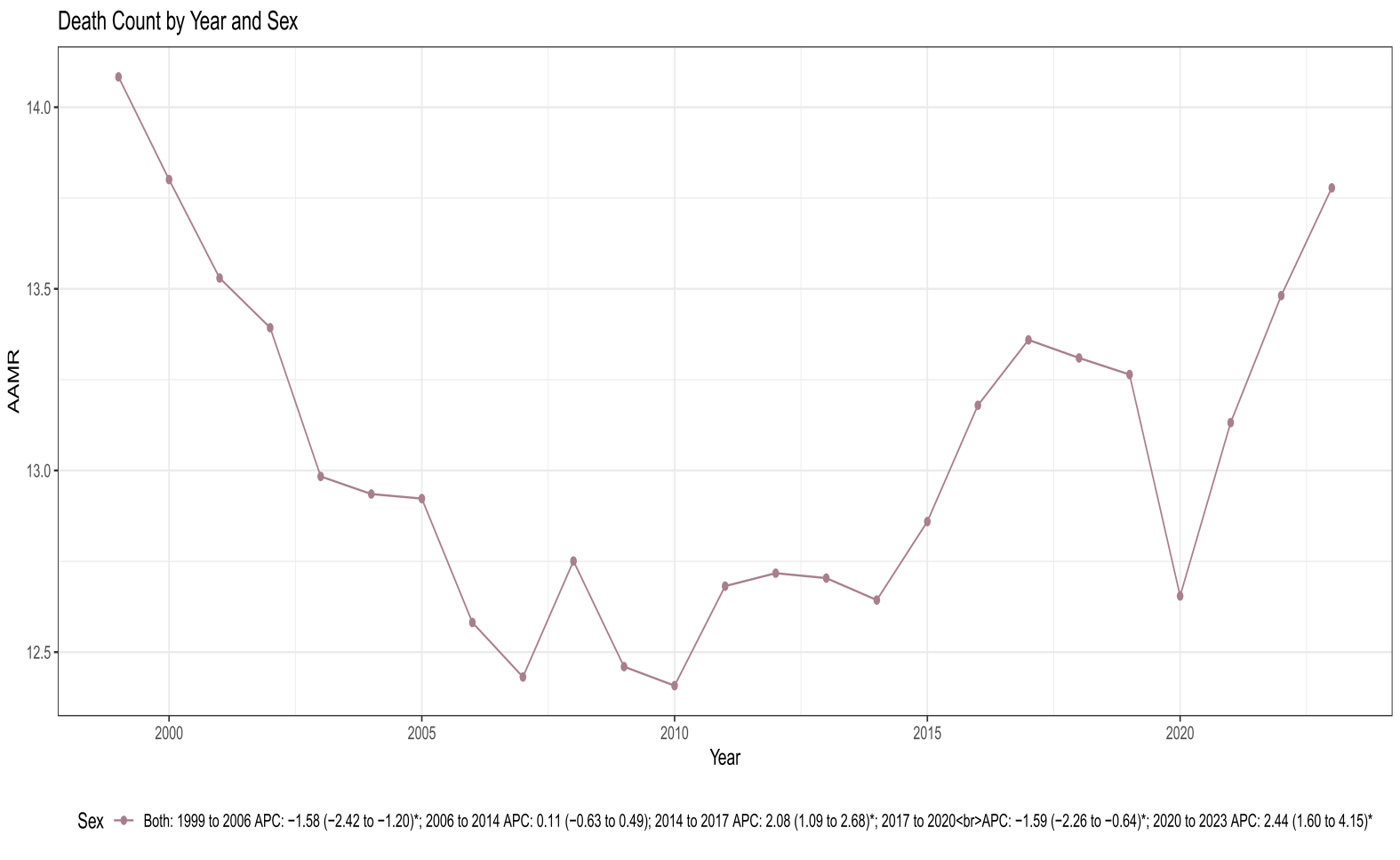

Supplement: Supplementary file 2 [file Table_1.docx]
